# (Self-)caring with companion animals: a qualitative exploration of how companion animals shape everyday practices of rest and relaxation

**DOI:** 10.1080/17482631.2025.2606862

**Published:** 2026-02-22

**Authors:** Renelle McGlacken, Vanessa Ashall

**Affiliations:** a Waltham Petcare Science Institute, Mars Petcare Inc., Melton Mowbray, United Kingdom

**Keywords:** Companion animals, self-care, rest and relaxation, obligation, wellbeing, multispecies

## Abstract

**Purpose:**

This paper aims to extend and enrich the concept of self-care, through demonstrating how self-care can manifest as both a relational and multispecies endeavour.

**Method:**

We qualitatively examined how people understand their relationships with companion animal(s) as shaping their practices of rest and relaxation. This paper is based on a reflexive thematic analysis of 31in-depth interviews with cat and dog owners across several countries.

**Results:**

Our analysis illustrates how practices of caring for companion animals and the obligations that drive them can also work to promote self-care. We draw out the ways in which obligations to care for companion animals can provide a sense of permission for and prioritisation of moments and activities that are experienced as mutually ‘care-full’. The paper reveals the role of companion animals in daily navigation and negotiation of temporal and normative barriers around rest and relaxation.

**Conclusions:**

This paper emphasises the important role that non-human animals can play in everyday practices and experiences of rest and relaxation.

## Plain language summary

This study looked at how people’s relationships with their pets affect how they rest, relax, and care for themselves. The researchers did 31 online interviews of people who lived with at least 1 cat or dog: 25 women and 7 men, with 1 interview involving a male and female couple. These people lived in several different countries across the world. The researchers looked for common themes across the interviews. The study found that caring relationships with pets had special, shared benefits for owners’ self-care and wellbeing. Needing to care for their pets gave people permission to take breaks and prioritize activities that benefited their own well-being, such as dog walks and petting. Pets also enjoyed these activities, which in turn enhanced the owners’ enjoyment. The results suggest that caring for their pets can help people to care for themselves through helping to manage time pressures and guilt which can prevent self-care. By enabling them to rest and relax, pets can help their owners to manage everyday stress and create wellbeing.

## Introduction

Self-care is a diverse and multi-dimensional concept (Godfrey et al., 2011), with its precise meaning and manifestation shaped by context. Taking a broad definition, the World Health Organisation (WHO) describes self-care as the “ability of individuals, families and communities to promote and maintain their own health, prevent disease, and to cope with illness—with or without the support of a health or care worker” (WHO, 2024), they advocate for widespread self-care “competency” (WHO, 2022).

However, contemporary rhetoric around self-care has received wide-ranging critique for being hyper-individualised and depoliticised, ignoring sociopolitical determinants of health, responsibilising citizens for their own care, and obscuring our interdependencies (Jones, 2018; Michaeli, 2017; Ward, 2015). In recognition of the self as “fundamentally social”, Letak, (2025a, p. 64) advocates for reconceptualising self-care as “social-self-care”. Relatedly, Rosset et al. (2024) highlight the positive wellbeing impacts of “other-oriented” acts such as caring and argue for an other-oriented model of wellbeing. This aligns with empirical work which highlights how, when asked about “self-care”, participants frequently describe it collectively, with acts of caring for others “often framed as a form of self-care” (Medina-Perucha et al., 2023, p. 4). What has yet to be explored empirically is the extent to which companion animals, rather than human companions, may contribute to their owners’ experiences of social and other- oriented self-care.

Studies have long examined the multiple ways in which companion animals can affect human health and wellbeing (Fine et al., 2025) and relationships with animals are said to make “a unique contribution to human wellbeing” (Riggs et al., 2024). With regards to their role in practices of self-care, interactions with and the presence of companion animals are often hypothesised to have a “stress-buffering” effect, mitigating the psychological and physiological impacts of stress. Such studies have examined how experimental (e.g., Polheber & Matchock, 2014) and naturalistic (e.g., Gnanadesikan et al., 2024) interactions between people and companion animals affect stress biomarkers such as cortisol, blood pressure, and heart rate, as well as psychosocial measures of stress (for a helpful overview see Harvie et al., 2025, pp. 545-547).

However, the establishment of an unequivocal buffering effect has generated mixed findings in the literature (Allen, 2003; González-Ramírez et al., 2018; Harvie et al., 2025; Janssens et al., 2021; Ogata et al., 2023). Similarly, studies of how pets affect human wellbeing more broadly present inconsistent findings (Scoresby et al., 2021; Martins et al., 2023; Ellis et al., 2024). Rather than indicating a need for further standardisation of methods and measures to conclude a generic “pet-effect”, McGlacken et al. (2025) argue for an embrace of this complexity through attending further to the relationality of pets and people and the ways that experiences of wellbeing are made through their interrelationships.

Our study responds to a lack of both in depth qualitative studies and engagement with the *practice* of caring as a way of making and doing daily wellbeing within literature on the role of companion animals in human health and wellbeing. We suggest that the specific social mechanisms and everyday practices which underpin the proposed “stress buffering effect” of companion animals, and its variability, could be better understood through such work. In doing so, we focus on rest and relaxation as daily practices of self-care, exploring how these can be facilitated by caring for others, namely companion animals.

Some aspects of relaxation and rest have previously been included and observed within studies of the impact of relationships with companion animals on human wellbeing (e.g., see Lea et al., 2024; Young et al., 2020). Other studies have explored how the types of activities engaged in with companion animals can affect different aspects of wellbeing and affectual states, with certain pet-related activities such as exercising and sharing household activities with dogs (Barcelos et al., 2020, p. 4) and engaging in tactile and sensory interactions with cats (Ravenscroft et al., 2021, p. 85) lending to feelings of calmness and relaxation. Yet, *how* relationships with companion animals shape rest and relaxation deserves specific theoretical exploration. In this paper we consider how companion animals support specific practices of rest and relaxation through the lens of care.

Indeed, broader scholarship has begun to take seriously the practice of care as a more-than-human endeavour, disrupting visions that conceive of “human survival and wellbeing independently from the rest of Earth’s beings’ (Puig de La Bellacasa, 2017, p. 140). Letak (2025a, p. 64) acknowledges that “taking care of animals and tending to the earth through activities like farming and gardening’ can represent important acts of self-care when the concept is “reframed to appropriately appreciate our sociality and relationality’. This echoes empirical studies of self-care experiences during the COVID-19 pandemic, with analysis showing how students in the Philippines understood caring for their plants and animals as a collaborative endeavour (Roxas, 2023) and residents in Spain described a sense of “mutual care and gratitude towards animals’ (Medina-Perucha et al., 2023, p. 8). Following this, we propose that by thinking about self-care as social, interwoven with giving care to and receiving care from known and unknown multispecies others, we can seek to better understand the care relations that support self-care. Yet, the practice of self-care in everyday life has received limited sociological attention to date (Letak, 2025b), with the role of human-animal (self) care relations in particular warranting further study.

Attending to this area, this paper conceptualises relationships shared between humans and companion animals as based in care. This directs us to consider how caring for companion animals plays out in relation to care for the self, focusing specifically on how caring for a significant non-human animal other might support or challenge experiences of rest and relaxation. We demonstrate how obligations to care for companion animals can provide a sense of *permission for* and raise the *priority of* rest and relaxation, with interview analysis indicating how caring for companion animals can provide routes to caring for the self. Our empirical insights reveal how self-care is navigated within sociocultural contexts where normative and temporal barriers may prevent the taking and making of time for the self. Illuminating the role of companion animals here provides important recognition of the significant roles that multispecies relationships can play in shaping understanding of the self and the practice of being well. In doing so, this paper aims to enrich and extend the concept of “self-care’ by locating it within intimate multispecies relations, illustrating self-care as both a social and more-than-human endeavour.

## Methods

This study aimed to explore the role of companion animals in human experiences of rest, relaxation, and sleep within the context of stress management. This paper is based on an analysis of rest and relaxation as practices of self-care which are shaped through care-based relationships with companion animals. To explore these areas, from September 2024 to January 2025 we undertook thirty-one in-depth semi-structured interviews with cat and dog owners across eleven countries. This method was chosen to centre participants’ own understandings of their relationships with companion animals, enabling them to describe the meanings they attribute to these relationships and the ways these can shape individual practices of rest and relaxation. As other studies have demonstrated, qualitative methods are particularly valuable for exploring relationships with companion animals (Charles & Davies, 2008). In health research specifically, insights into the significance of relationships shared with companion animals can emerge in interviews, even when not included as an explicit part of the research focus (Ryan & Ziebland, 2015).

### Ethics

The study received institutional ethical approval from the Mars Research Review Board at the Waltham Petcare Science Institute (approval no: 202412607). Participants were recruited via several rounds of circulating the recruitment call on social media platforms, pet care networks, the research team’s own networks, and “snowballing” via participants. This involved sharing a project summary and flyer with a link to a screening survey which asked those interested to give further detail on the companion species and number owned, as well as demographic information regarding their own age group, gender identity, country, and an email address. After completing this brief survey, the participant information sheet and consent form were shared via email and potential participants were given the opportunity to ask any questions about the study. On receipt of signed consent forms, online interviews were arranged at a convenient date and time.

### Participants and interview setting

Twenty-five interviews were undertaken with women and seven with men, with one interview involving two participants - a male and female couple. Participants ranged across age groups and geographical locations (including the UK (12), US (4), Australia (4), France (3), Canada (2), Mexico (2), Germany (1), New Zealand (1), Malaysia (1), China (1), and Kazakhstan (1)). All participants lived with at least one dog or cat, with many participants living with multiple of either or both species.

Interviews were conducted online by the first author using Microsoft Teams and Zoom video-conferencing software. Interviews ranged in length from the shortest at twenty-eight minutes to the longest at seventy-one minutes. A semi-structured interview guide was used which began with an overview of the study and the interview structure. Interviews opened by asking participants to “tell me a little bit about your pet(s)?” and worked through four key sections relating to relaxation, rest, sleep, and broader management of everyday stress. Interviews closed with an opportunity for participants to introduce additional topics or return to a topic. Each thematic section worked through participants’ understandings of the topic (e.g., “what does “rest” mean to you?”), the role they felt their companion animal(s) played in this, and the importance of this topic in their relationship with their companion animal.

During interviews, many participants shared stories which were emotional (e.g., the loss of a past companion animal or challenging times shared together). To navigate these instances, the lead researcher drew on Whitney and Evered's (2022) Qualitative Research Distress Protocol (QRDP) to provide reassurance and, as appropriate, the option to stop for a moment, move on from a topic, or end the interview. No participant chose to leave an interview early due to emotional distress. On closing, many participants expressed appreciation for the opportunity to speak at length about relationships shared with their companion animals and the role this plays in their life. Such instances emphasise the importance of qualitative methods in this area of study, enabling participants to express in their own words the significance of their relationships with companion animals in relation to their everyday wellbeing.

### Analysis

After being professionally transcribed, the interview data was uploaded onto the qualitative data analysis software NVivo 15 for better functionality in handling and coding. To analyse the interview data, we adopted Braun and Clarke's (2022) reflexive thematic analysis (RTA), moving through the non-linear steps of familiarising with the data, coding, and developing overarching themes through which to make sense of the codes. In doing so, we followed Byrne's (2022, p. 1396) experiential approach to RTA, also wanting to “emphasise meaning and meaningfulness as ascribed by participants”. Like Byrne, although we understand the relationships and practices at the core of this study as socially constructed and emerging in relation to each other, we aimed to centre participants’ own experiences rather than critically examine the making of such constructions. In taking a reflexive approach to thematic analysis, we acknowledge our role in creating the themes from our interpretation of the data and positioning around this topic, seeing these as generated rather than emerging and “infused” with our subjectivity (Braun & Clarke, 2022, p. 4). Importantly, we began from a standpoint which takes seriously the significance of relationships shared with companion animals and the research was doubtlessly shaped by both authors’ own experiences of living with pets.

Throughout the analysis, which we define as socially constructed, we remained reflexive about our positionality as researchers, and the broader social contexts of the participants and their pets. For example, it is notable during our analysis that many participants voluntarily discussed working from home when exploring the role of their pets in rest and relaxation. Whilst this was not the focus of our study and participants were not selected on this basis, it is interesting to consider whether this could represent an unexpected sample bias (given that interviews were held online and therefore perhaps easier for hybrid or remote workers), or whether pets may be differently involved in rest and relaxation for this group. We are also aware, both as pet owners and researchers, that positive experiences of pet ownership are not fixed, and that our participants may not have explored some of the more challenging aspects of pet ownership and their consequences during the interviews. We acknowledge that our positioning as researchers working within the pet care industry may have led to a social desirability bias, with participants feeling that reflections on negative or difficult aspects of their relationships with companion animals were not as welcome as positives. Nevertheless, we remained open to exploring challenges discussed within our analysis.

Interview data was coded around key headings of pet acquisition and background, relaxation, rest, sleep, stress, and wellbeing. Coding involved generating a mix of more simple and more complex descriptive codes which captured what people and their pets are described as doing and creating e.g., feeding, stroking, walking, companionship, routine, and responsibility. Thinking about what these things are accomplishing in the context of rest and relaxation, with caring identified as a prevalent theme across the dataset, the next stage of coding connected these with more complex analytical codes such as “obligation” and “justification”. Bringing together our understanding and interpretation of what, how, and why in participants' accounts, codes around “routine”, “time”, “leisure”, “responsibility”, and “acceptability” became subsumed under broader themes, i.e. “granting permission” for and “prioritising” rest and relaxation.

## Findings

Data analysis presented in this paper is organised into two key themes. The first theme “Granting permission and prompting prioritisation” examines the key processes via which relationships with companion animals are understood as shaping participants’ experiences of rest and relaxation—the “*how”*. Our second theme “The role of mutuality” explores the grounds that underpin these processes—the “*why”*.

To protect anonymity, participants are referred to by their interview number throughout and names of companion animals have been redacted. Being mindful to retain context, ellipses within square brackets have been used indicate where interview quotes have been trimmed for brevity.

### Taking and making time: granting permission and prompting prioritisation

Across the interviews, many participants discussed ways in which their dogs and cats enable, encourage, and enhance their experiences of rest and relaxation in ways which indicated the importance of two different but overlapping processes: granting permission and prompting prioritisation.

Relating to the former, participants often described how their companion animals helped them gain internal and external permission to engage in rest and relaxation activities because these were undertaken as a required part of pet care. This was often connected to the nature of such activities as obligations or responsibilities, shifting the meaning of these activities from “leisure” and self-directed to a fulfilment of caring duties. As the following participant describes:

“I think I see—walking the dogs as a kind of responsibility. It has got to be done. It's on my list of jobs, (Laughs) almost, for the day, whereas if […] the option was to, say, go out for a walk by myself, go for a jog by myself, or go out on my bike. That would be seen as more of a leisure activity, in my mind. I think I”d feel guilty doing that during the day, during the working week, […] this is more like a responsibility. It has got to be done. It's on my list. (Laughs)”

(Participant 6, woman, 45-54, multiple dogs, UK, living with pets only)

Here, the framing of dog-walking as a “responsibility”, a “job”, and something that “has got to be done” moves the walk itself from being primarily a *leisure activity* to an *obligation*. For many participants, walking with and *for* their dogs is suggested to mitigate feelings of guilt that would be attached to taking a walk by and for themselves. In this way, care duties towards companion animals may not only prompt rest and relaxation activities, but also enable people to engage in them.

There were clear and unsurprising species differences in the types of pet-related activities which pet owners experienced as restful or relaxing. Whilst dog owners often described involvement in physical activities such as walking, pet cats contributed to the theme of permission and prioritisation through more sedate and indoor activities. For example, when discussing the role that their cats play in their experience and management of everyday stresses and anxieties, one participant described how they benefit from the general presence of their cats and seek out tactile interactions with them when they need a break from work:

“I’ve noticed it since, like with a lot of people with the pandemic starting to work from home and stuff, just like having my cat on the desk with me or whatever. There's a stressful moment. I would say work probably causes the most stress for me. Just having them there, like, you can pet, like even if you're not even thinking about it actively as, like, they're there to calm you. Sometimes I’ll just get up and go find one because I just need a break, but they're definitely part of the stress management, for sure.”

(Participant 8, woman, 35−44, multiple cats, US, living with spouse/partner and children)

When asked whether they would feel able to take a similar break from work if they did not have the cats around, the participant explained that it feels “more acceptable” to take breaks to spend time with companion animals:

“I don't know that I necessarily could. I guess I would have to find something, but I also feel like it's more acceptable, I guess, in a way, to take a break and go be with your pet, than it is to, I don't know, go out for a run in the middle of the day or whatever. (Laughter)”

(Participant 8, woman, 35−44, multiple cats, US, living with spouse/partner and children)

The contrasting feelings of guilt and acceptability identified by owners of both species highlight how companion animals can be understood not only as providing a variety of activities which are experienced as breaks (e.g., walking the dog or stroking the cat) but, importantly, pet care also provides a more legitimate reason to do them.

Overlapping with this suggested sense of permission are the ways in which companion animals can also prompt a *prioritisation* of activities and moments for rest and relaxation. A key aspect here is the connection made between prioritising the care of companion animals and self-care. On this point, many participants articulated narratives around how their companion animal stimulates a different relationship with time. For example, it was reported that specific times of day were utilised in more restful or relaxing ways due to pet care commitments:

“[...] I started to prioritise moments of the day. So, before, I was like, “Oh, I will,” I don’t know, “eat in front of my screen, and I will work,” or, “I will work at the end of the day, quite a long time,” because, well, kind of, there’s no rush. […] But, when you have a dog, first and foremost, he forces you to go outside, which of course is amazing, just breathing. And even now, he’s my big switch. So, when I end the day, I’m like, “I’m closing the laptop and I’m going with him for one hour.” And I sometimes even don’t take the phone with me, nothing. So, I just walk and enjoy time with him.”

(Participant 13, woman, 35−44, one dog, France, living with spouse/partner)

This participant describes a situation prior to acquiring their dog in which time for the self—“me time”—was not being prioritised and they were experiencing a lack of balance between work, leisure, and rest. They suggest that their dog has helped change this dynamic by prompting a making and taking of time outside of work via dog-walking duties and a more present relationship to this temporal experience.

Another way in which the theme of prioritisation was experienced was through a more positive perspective on life that pet care responsibilities can generate. In this case, the dependency of companion animals is suggested to balance out the significance of work and other stresses, through putting them in perspective, for example:

“Before it was only about me (Laughs), like me in my life selfish, like no one depends on me but now that I have this creature depending on me, I feel like I feel way more compelled to just enjoy and just say, “Okay, you know, putting everything in a balance.” Saying, “Okay, work is important, but I mean work will survive without me. My kitten will not. So, I really feel way more de-stressed.”

(Participant 4, woman, 25−34, one cat, France, living with spouse/partner)

The dependency of companion animals helped with the prioritisation of rest and relaxation because caring for companion animals was often expressed as not requiring self-driven motivation in the way that other daily tasks might. Dependency was discussed as both driving the prioritisation of caring tasks as well as imbuing them with personal enjoyment and reward, for example:

“I think it”s having something, someone beyond myself to care for. I feel like if I’m really down or just not in a good place, I don’t care so much about how I’m doing, but I don’t want to let other people down and so if I am held accountable by some other living thing, that’s not a plant because a plant isn’t enough. Having a pet, having a spouse, somebody that I see every single day, that’s what makes the difference for me.”

(Participant 18, woman, 35−44, multiple dogs, US, living with spouse/partner)

Dependency and obligation in companion animal care led to the discussion of inflexibility and necessity, although these were often spoken of positively in terms of the role that caring obligations towards companion animals can play in structuring everyday life. For example, dog-walking was discussed by multiple participants as something they enjoyed or felt was good for them, but would find more challenging to prioritise if it were just a walk done for themselves:

“I have to take her out and get outside, and I always feel better, once I’ve gotten outside. But, it’s like, I don’t know that I would, without a dog being like, “You need to do this,” it’s harder to make that a priority.”

(Participant 22, woman, 25−34, both dog(s) and cat(s), US, living with companion animal only)

The notion of personal responsibility was explored in terms of discipline by some participants, where this responsibility was more easily directed towards pet care, rather than self-care:

“I think, one of the things is discipline, that I go out. Not quite every day, but I would say I might miss out one day a month over the year, through bad weather or other things I'm doing, but I have to get and take her out for a walk. We usually go out for an hour and a half in the local park. She really enjoys it, and it has become my job, my duty. So, it gives me a bit of discipline.”

(Participant 24, man, 65+, one dog, UK, living with spouse/partner)

As such, participants framed both active and passive benefits of pet care inflexibility in forcing a prioritisation of activities they know they benefit from but find hard to do for themselves. The value of inflexibility in pet care appeared to relate to some extent to a lack of self-motivation participants feel in prioritising their own rest and relaxation:

“So I think having the dog, in particular, helps with I know I have to go out at some point in the day […] It takes a bit of the weight off of having to create that for myself because, yeah, as I say, you’re doing it for a purpose rather than… it’s a purposeless thing.”

(Participant 23, man, 25−34, one dog, UK, living with spouse/partner)

Furthermore, for this participant, a walk without their dog is reflected back on as “a purposeless thing”, whereby the purpose, and thus experience, of the walk become changed when done with and for their dog.

Yet finding the time for care-activities, particularly dog-walking which was often described as a daily necessity, can also be challenging and multiple participants discussed how accommodating this when time was limited could also generate stress. Interestingly, although relaxation derived from dog-walking can be complicated by other demands for time, the compulsory framing of the dog-walk can still be reflected upon as forcing balance and breaks amongst other competing priorities because they seem of lesser urgency retrospectively. For example, one participant described how when undertaking their PhD: “[my dog] imposed on me to rest, which is a really good thing”, however, in further discussion, different attitudes towards these forced breaks are drawn out:

“I didn’t have a choice: I needed to go out with her, so it allowed me to take some break. But, sometimes, of course, it was stressful, because I thought I didn’t have this time. So, I had to do it, which now, I think it’s a good thing, but at this time, I was, “Oh, no, no, I have so many things to do, and in addition, I have to walk [my dog].” So, this can be stressful. But, if I’m stressed, she offers me so much distance from my problems, that if I do the balance, it’s good to have her by my side, because she’s always super happy, super calm. As I said, she’s an example to follow for me, so she helps me.”

(Participant 5, woman, 25−34, one dog, France, living with spouse/partner)

Such insights signal the importance of context in our analysis, demonstrating that the ways pet care activities fit into everyday routines and relate to other time commitments shapes experience as (un)relaxing, even if they are overall valuable in retrospect.

In this section, we have presented how obligations to care for companion animals can help justify and raise the importance of breaks and leisure-related activities. When connected to caring for companion animals, rest and relaxation activities were described as more permissible and a higher priority, with the provision of care for companion animals thus providing an indirect route to practicing self-care.

### Sharing is caring: the role of mutuality

The emotional underpinnings of the role that caring obligations towards companion animals can play in supporting self-care is the *mutuality* of sharing rest and relaxation experiences with companion animals. Going beyond the shared physical and mental benefits derived from care-based activities that participants do with their companion animals, mutuality signals how people also benefit *emotionally* from an enjoyment of the animal’s perceived enjoyment. Enhancing the functional benefits of pet-related activities are the *relational* benefits of doing things together:

“If I go for a walk without her, okay, it’s good to walk for me, but if I go for a walk with her, I go for a walk, so it’s good for me, and, in addition, I make good for someone else, and it’s amplifying my happiness.”

(Participant 5, woman, 25−34, one dog, France, living with spouse/partner)

Here, going out for a walk with their dog reflects an act of goodness for themselves and their companion, thus “amplifying” the enjoyment derived from it. As such, the connection shared between people and their companion animals appears to be key to the mutual benefits of caring:

“I’ve got other hobbies, I love crafting and things, so that brings me some joy and switches stress off. But not in the same level that they do, I think, because you have to be non-selfish when you have them, and you have to spend time with them one-on-one. And you get the love back from them and you don’t get that from anything else. You just get that love back that you know that their world revolves around you and that’s quite a nice feeling some days when you’re like, oh, have I got a purpose? What am I doing? And then you turn into mum mode and deal with them instead. So it’s quite nice.”

(Participant 11, woman, 35-44, multiple cats, UK, living with spouse/partner)

For this participant, unlike other hobbies that they enjoy doing for rest and relaxation, spending time with their cats involves multiple relational aspects: “one-on-one” connection, the giving and receiving of love, the responsibility to care, and feelings of purpose.

Importantly, the derivation of personal happiness from the happiness of companion animals was important for many participants when describing why they felt they benefited from spending time with their companion animal in regard to rest and relaxation:

“I just really enjoy having her around. And so yeah, she makes me happy and also, you know, just knowing that I’m there for her, as well, during that time because I'm away so much. So yeah, just giving her something, as well. So, it’s not only she, like she’s there for me to make me happy but also, yeah, just knowing when I’m there for her, I know that she’s really happy.”

(Participant 15, woman, 25−34, one cat, UK, living with spouse/partner)

Again, the theme extended across species, with more energetic interactions often being described as mutually enjoyable by dog owners:

“[…] the joy on her face when she was exploring the woods, because it was no boundaries there. She'd leap off and then, five minutes later, she'd reappear from somewhere, but the excitement and the amount of pleasure, it rubs off […] give happiness to something else. It's therapeutic for her—for us, as well.”

(Participant 24, man, 65+, one dog, UK, living with spouse/partner)

Our analysis indicates that rather than affecting owner rest and relaxation and general wellbeing in a transactional or instrumental sense, spending time with companion animals can benefit people because it provides an opportunity to care for a significant other and enjoy their enjoyment.

A particularly interesting finding was that beyond the mutual enjoyment of pet care activities e.g., walking, grooming and play, other everyday relaxation and leisure- activities which can be enjoyed without pets were also described as enhanced when engaged in with companion animals:

“I don’t know how to say it, like a base or an extra sensation of having her… because, some people may go and relax with just watching TV or just going for a walk, but having her, it’s another layer of protection or help with the mental health, I guess. Because, even if I take her for a walk, because I want to be a responsible owner, but at the same time, I want to be enjoying the walk, I try to avoid using my mobile phone while I’m walking with her […] So, in a way, it takes my brain, my mind out of whatever I should be thinking of. And she loves to roll on the grass as well, so I love to see that. So, again, it’s another layer of just going for a walk and see the birds.”

(Participant 16, woman, 45−54, one dog, Canada, living with spouse/partner)

Here we note that everyday activities such as watching television as well as petcare activities such as going for a walk are changed from the status of “just”, being lesser in value or quality, to something deeper and richer when shared with companion animals. The participant’s metaphor of “layering” beautifully expresses a key element of this theme, whereby participants described how their companion animal adds to, builds on, or extends their experience of a wide variety of activities which may be expressly undertaken for rest and relaxation, but with the enhanced benefits of mutual enjoyment providing “another layer of protection or help with the mental health”.

This recognition of the potential for mutual benefits from both pet care and non-pet related activities also highlighted a risk, whereby if the companion animal was not enjoying an experience, their owner could also not enjoy it fully:

“I would say, in terms of things that make it more relaxing, the fact that if I am sitting… if I get to the end of the day, have my tea, put the television on and sit down, the dog will come and sit either adjacent to me or lying across me. And it is a very comforting thing. It”s like, “We’re settled now. We’re not going anywhere. We’re not doing anything.” It’s like you can fully unwind. And on the flipside of that, actually, when he’s not settled… like, there have been fireworks recently and he was very agitated and up and about, that… it wasn’t that it was deeply unrelaxing, but it did interrupt that regular flow of winding down in the evening because he’s unsettled. So you start to realise him being relaxed as well as me is quite a key dimension of my evening routine.”

(Participant 23, man, 25−34, one dog, UK, living with spouse/partner)

As this participant explains, in the first scenario, their dog’s presence provides comfort and a capacity to “unwind” to a greater extent, even when watching TV. In the second scenario, when their dog seems unsettled, although the fireworks themselves were not necessarily disruptive to the experience of watching TV, their impact on the dog meant that the shared “flow of winding down” is disrupted. Again, such insights illustrate how engagements with companion animals are not instrumental, owners did not describe engaging with their animals as a resource designed to provide relaxation or other wellbeing outputs. Rather, our analysis reflected the multiple ways in which humans and companion animals can together build routines and atmospheres which support mutual rest and relaxation but also require it.

Overall, mutuality helps us understand *why* sharing activities and time with companion animals is beneficial in the first place. Whilst relaxing and restful activities are often in themselves of mutual benefit, more than this, mutuality also represents the enhancement and enrichment of these activities. Companion animal owners described how rest and relaxation-related activities are enjoyed more with companion animals because of a *sharing* of benefit. In this way, the relationship between companion animals and owner rest and relaxation is not instrumental, but deeply relational and dependant on a collective rather than individual enjoyment.

## Discussion

In the introduction we described this paper’s intention to enrich and extend the concept of “self-care” by locating it within intimate multispecies relations, illustrating self-care as both a social and more-than-human endeavour through examining the rest and relaxation practices of pet owners.

Our data analysis identified two key processes by which companion animals were understood as enabling and motivating rest and relaxation: permission and prioritisation. Regarding the former, companion animals were described by multiple participants as providing a sense of permission and justification to engage in activities related with rest and relaxation because they were reframed as care duties for a dependent and significant other.

Our analysis showed that intimate relationships with companion animals can alter the meaning of certain activities and the broader notion of a “break” from being something self-directed and within the realm of “leisure”, to a fulfilment of caring responsibilities and therefore within the realm of “care-work”. In this way, self-care through the care of companion animals can be seen as providing routes to navigate norms and expectations around leisure, breaks, and rest and relaxation.

For our participants, the aspect of time is significant, shaping relationships to and experiences of activities associated with both caring and rest and relaxation. Centring the moral ordering of modern Western relationships with time, Snyder (2013, p. 244) argues that clock time has become a “moral institution' that informs how we construct meaningful activity. Key here is a “culture of busyness” that dictates a routinised structuring of daily life, with time filled by activities that involve a focusing of energy (ibid, p. 258). Being affected by, but consciously outside of this culture, our analysis raises questions around whether companion animals can break up schedules and interrupt the ordering of our days in ways which promote different relations with time.

Overall, that care obligations towards companion animals are indicated to provide a sense of permission to engage in activities associated with rest and relaxation reveals the everyday negotiation of norms and expectations around managing time. Although spending time with companion animals will rarely be experienced as purely obligation-based, in the context of personal practices of rest and relaxation, the obligation to care for companion animals can provide an important justification for taking time for activities that provide care for both our animals and ourselves. In this way, care duties towards companion animals may not only prompt rest and relaxation activities, but also enable people to engage in them.

Returning to our introductory literature, we suggest that our findings support previously identified contemporary understandings of self-care as a social activity (Letak, 2025a), and demonstrate the possibility of animal, as well as human other-oriented wellbeing practices (Rosset et al., 2024). For example, our analysis revealed the ways in which the prioritisation of companion animals can help people prioritise activities that they may find difficult when done by or for themselves, when they may have the flexibility to *not* do them. Acknowledging the self as emerging through relations with other beings, things, and contexts, Letak (2025a, p. 64) considers that through caring for others we often “fill our own cup”. Self-care is dependent on networks of known and unknown others and performed via webs of relation, with the meaning and manifestation of self-care shaped by our particular lived contexts. In our case, companion animals can now be understood as helping people prioritise the doing and making of time for activities which they feel are “good” for them but often become deprioritised. This raises important broader questions about experiences of practicing self-care, with self-care felt as difficult to prioritise for some due to negotiating other obligations and priorities or experiencing a lack of “self-care worthiness” (Furlong and Wuest, 2008).

Our analysis has indicated that in cases where people struggle to care about themselves, caring for others that we care about—here pets—can help, e.g., through providing a sense of purpose and other-oriented motivation. This aligns with Rosset et al.'s (2024, p. 10) advocacy for other-oriented wellbeing interventions, showing how those who “have a harder time helping themselves than helping others’ may also help (or care for) themselves by helping (or caring for) non-human others. Yet, although companion animals may help people navigate the rocky terrain which complicates caring for themselves, their role in doing so highlights the persistence of problematic norms and structures which undermine our permission for and prioritisation of self-care in the first place.

In our analysis, we focused on obligation as a key concept through which other oriented self-care was manifested across species. Here, we are concerned specifically with “role obligation” (Barbalet, 2020), considering how the role of guardian/owner can evoke care duties towards companion animals (Glanville et al., 2020; Hens, 2009), which can then justify and encourage forms of self-care. Previous studies have confirmed the important role that care (Degeling & Rock, 2013) and obligation (Westgarth et al., 2014; Westgarth et al., 2017) plays in motivating dog-walking. It is pertinent that particular activities undertaken for the wellbeing of companion animals can also align with activities we might engage in for our own wellbeing, such as the physical, emotional, and social benefits experienced through dog-walking (Johnson et al., 2011).

Obligation is important in our study because companion animals were discussed as influencing how rest and relaxation are negotiated in relation to norms and expectations around work, chores and leisure. Like Junça-Silva's (2023, p. 2) finding that human-animal interactions can provide teleworkers with “micro-breaks” that help to “recover their regulatory resources by making them experience relaxation and calmness, and control […] and distracting them from work”, participants in our study also described how interactions with companion animals can provide both planned and spontaneous breaks from work and its associated stresses. Going further than the work context, it is also important to recognise in our analysis the ways that companion animals can structure and enrich daily life, providing routes to and further enjoyment of leisure time. Introducing the concept of “everyday moments of leisure”, Gallant et al. (2024, p. 251) argue that such moments have potential for supporting wellbeing, challenging the assumption “that the ordinary is not meaningful”. Our study suggests that companion animals can play a significant role in creating opportunities for and deepening enjoyment of an everyday “multispecies leisure” (Danby et al., 2019).

Ultimately, this analysis raises important questions for individualised narratives around self-care. The role of companion animals in prioritising activities recognised as beneficial but deemed less important when done only for the self perhaps indicate that framings of self-care may be more salient when treating the self as relational, rather than individual. A key contribution of our study is that these self-care relationships can exist across species. In playing into the ways our wellness is caught up with others, how the happiness of those we love feeds into our own, self-care initiatives may be more relevant and effective when taking account of the relationality of wellbeing (White & Jha, 2023), and we propose that this should include a consideration of multispecies wellbeing (McGlacken et al., 2025). Whilst it remains important to keep the individual in view as deserving of self-care, we can also recognise their intertwinement with others, including companion animals, and the ways that care, even towards the self, can be practiced as a social activity.

We highlighted earlier a lack of in-depth qualitative research which examines the significance of companion animals for human health and wellbeing, particularly those which attend to the role of care. By using qualitative methods to explore participants’ own meaning-making about the specific practices through which companion animals shape their everyday experiences of health and wellbeing, this analysis has provided rich insights into how people and companion animals make everyday routines and moments for rest and relaxation together. This work expands beyond measuring the effects of animal presence or specific interactions which have been the key focus of many previous studies in this area (Allen, 2003; González-Ramírez et al., 2018; Harvie et al., 2025; Janssens et al., 2021; Ogata et al., 2023). In this way, this paper has shown that rather than providing a clear and measurable “stress buffering effect”, companion animals may help people to practise rest and relaxation, which allows for management of everyday stresses and the creation of daily wellbeing.

Our qualitative approach has also allowed us to explore what complex phenomena such as wellbeing mean to participants themselves. For instance, in allowing participants to describe what activities such as dog walks *mean* in relation to rest and relaxation, our analysis extends understandings of their physical, mental, and social benefits by highlighting how they are prompted, justified, and enjoyed through their embeddedness in care obligations. Overall, our qualitative approach has illuminated ways in which companion animals can structure and enrich daily life, providing routes to and further enjoyment of rest and relaxation.

A key feature of our analysis is the location of self-care within intimate interspecies relationships, in contrast to transactional framings of the health consequences of pet ownership. Core to the identified routes through which companion animals can shape experiences and practices of rest and relaxation is a sense of *mutuality*, that the activities engaged in for companion animals are also beneficial to their caregivers. Mutuality here involves a mutual benefitting from the justifying, prioritising, and doing of restful and relaxing activities and an enjoyment of their companion animal’s enjoyment. This relational underpinning reflects the importance of feelings of connection and love in participants’ everyday doings with companion animals.

Our analysis supports and develops contemporary understandings of multispecies and more-than-human care (Puig de La Bellacasa, 2017), through highlighting the significance of the nature of the companion animal relationship for self-care. For Lynch (2007), love changes the nature of care. In the context of primary care relations, those involving “strong attachment, interdependence, depth of engagement and intensity”, Lynch observes that “love labour” can arise as the “work required to sustain these relations” (Lynch, 2007, pp. 555–557). Though primarily directed by doing good towards the other rather than the self, love labour is often characterised by a “sense of mutual dependence” (ibid, p. 559). In this way, Lynch claims that love labour can provide “nurturing capital” to both the giver and the receiver. The identification of love labour across species in our analysis is useful for directing novel thinking around the role of companion animals in human health and wellbeing away from an instrumentalisation of such relationships.

As Riggs et al. (2023, p. 563) found in their Australian study of how companion animals affected interactions between young trans people and healthcare professionals, people may relate to animals in ways which “speaks to benefits” but are not rooted in purposeful “therapeutic animal-related goals”. In this way, relationships with companion animals may not be therapeutically driven but may still provide therapeutic benefits. Lynch makes a similar point in emphasising the non-commodifiable nature of love labour, asserting that there is “no clear identifiable project” for labours of love, with the goal instead being the relationship itself (ibid, p. 566). The mutuality theme in our analysis extends Lynch’s argument across species; although some participants might describe engaging in intentional interactions with companion animals for the relational support they can provide, the fundamental relationship is rarely described as one of sole utility.

Our findings have important practical implications in their support for a move beyond the recognition of the physical presence of animals as potentially therapeutic, for example in the case of therapy animals, instead bringing into focus the therapeutic potential of everyday relationships with loved animal companions. Emphasising their importance in supporting health and wellbeing, Brooks et al. (2016, p. 10) has contended that “pets should be considered a main, rather than a marginal source of support” in the management of mental health and other research has assessed whether healthcare professionals can “activate” companion animals to “positively impact social determinants of health” (Hodgson et al., 2018, p. 105).

Mutuality is central to our argument precisely because caring for others is not understood as inherently contributing to care of the self. For example, Britton et al. (2023) report how for diabetic women with care-giving responsibilities, caring for others can sometimes be exhausting and in tension with self-care. In contrast, the frequent alignment of things we might do to care for ourselves and the activities we typically undertake for the care of companion animals is important to acknowledge as a potentially unique aspect of our relationships with companion animals, particularly in the case of domestic cats and dogs. Although mutuality does not mean equality, the category of “companion animals” (or “pets” as a broader construct which spans both the animate and inanimate (Wrye, 2009)), perhaps suggests the mutualisms built into these special relationships through historical and ongoing processes of co-evolution and domestication (Sandøe et al., 2015).

### Limitations

It is important to recognise some of the limitations of this study. As a qualitative study, this research did not aim to make generalisable claims. Rather, the analysis presented here has illustrated common themes identified as significant to the topic of rest, relaxation, and management of daily stress in this dataset. Although interviews were undertaken with participants from a range of countries, these were mostly conducted with participants from Europe and Northern America. Whilst relationships with companion animals and practices of pet ownership vary within and across countries, it is likely that there are important cultural differences missed by this limited geographic spread. Similarly, alike with many studies of relations with companion animals (Rodriguez et al., 2021), our interview sample was skewed towards women. Given that men may experience mental health and practice self-care differently (McKenzie et al., 2018; McKenzie et al., 2025), it will be important for future work to explore potential gender differences. Finally, although companion animals represent a range of species, this study focused only on domestic cats and dogs. It was further limited by not specifically seeking to include participants whose companion animals were experiencing behavioural or health issues, which are recognised as having significant negative impacts on owner wellbeing (Barcelos et al., 2023). Although several participants discussed experiences with animal behaviour and health issues, not including this as a key component in our recruitment strategy may have shaped our dataset on rest, relaxation, stress, and self-care in important ways.

## Conclusion

This paper aimed to enrich and extend the concept of “self-care” by locating it within intimate multispecies relations, illustrating self-care as both a social and more-than-human endeavour.

Through providing empirical insights into the role of companion animals in human experiences of rest and relaxation, our analysis has drawn out detailed understandings of companion animals as helping owners navigate and negotiate temporal and normative barriers around rest and relaxation. When viewed through a multispecies care lens, obligations to care for companion animals were indeed described as presenting routes to self-care, enabling and encouraging people to engage in activities by providing senses of permission and prioritisation. We showed how these self-care mechanisms are, crucially, located within intimate multispecies relations, because underpinning this process are experiences of mutuality, with pet-related activities often experienced as mutually beneficial and further enhanced by being shared. In this way, companion animals not only help us *take* and *make* time for ourselves, but can also turn this into *quality* time, with the relational connection experienced when engaging in activities with and for companion animals enhancing their significance and enjoyment.

Beyond this paper’s significance in supporting the expansion of social and other-oriented understandings of self-care, including across species, recognition of the significance of companion animals in peoples’ lives is also important for both scholars and practitioners of health and wellbeing. This paper has provided further weight to this recognition, signalling key aspects that make relationships with companion animals special and significant in everyday practices of wellness and self-care.

Finally, through considering positive aspects of obligation, this paper suggests that not only do we have obligations to other animals in terms of promoting their positive experiences, but that we can also benefit from our responsibilities to do so. Accounting for self-care as a relational and multispecies endeavour, future work can fruitfully explore the role of humans and animals in shared networks of care, supporting us to care for ourselves through caring for each other.

## Data Availability

Interview data generated through this research is not publicly available due to ethical and privacy considerations. Participants did not consent to the public sharing of their transcripts, and the data may contain potentially identifiable information.
